# Linking Health Financing to Oral Health Coverage and Disease Burden in SEARO Countries: A Cross‐Sectional Analysis of Country Level Data

**DOI:** 10.1002/hpm.70027

**Published:** 2025-09-30

**Authors:** Shashidhar Acharya, Manu Raj Mathur, Santosh Kumar Tadakamadla, Angela Brand

**Affiliations:** ^1^ Manipal College of Dental Sciences Manipal India; ^2^ Manipal Academy of Higher Education Manipal India; ^3^ Faculty of Health Medicine and Life Sciences (FHML) Maastricht University Maastricht the Netherlands; ^4^ Bart's and the London School of Medicine and Dentistry Queen Mary University of London London UK; ^5^ Dentistry and Oral Health Department of Rural Clinical Sciences La Trobe Rural Health School Bendigo Australia; ^6^ Violet Vines Marshman Centre for Rural Health Research La Trobe Rural Health School Bendigo Australia; ^7^ Prasanna School of Public Health Manipal Academy of Higher Education Manipal India; ^8^ United Nations University—Maastricht Economic and Social Research Institute on Innovation and Technology Maastricht the Netherlands

**Keywords:** financing, oral‐health, SEARO, South‐East‐Asia, sustainable development goals, Universal‐Health‐Coverage

## Abstract

**Introduction:**

The Southeast Asian region of World Health Organization (SEARO) comprising 11 countries, that is Bangladesh, Bhutan, Democratic People's Republic of Korea, India, Indonesia, Maldives, Myanmar, Nepal, Sri Lanka, Thailand, and Timor‐Leste is home to a quarter of the world's population where severe oral health disparities persist.

**Aim:**

This study aims to collate the oral health financing landscape, evaluate the relationship between government health expenditure and the burden of oral diseases, assess the proportionality of oral health spending relative to its share of the overall disease burden, and examine the inclusion and funding of dental care within Universal Health Coverage (UHC) benefit packages in the SEARO region.

**Materials and Methods:**

Data for this study were sourced from publicly available databases and relevant national health statistics repositories of SEARO countries. These datasets provided information on health financing indicators, oral health coverage, and oral disease burden. Descriptive statistics were used to summarize indicators across SEARO countries. Correlation analyses were done to examine the interrelationship between health financing indicators and oral health outcomes and oral health coverage.

**Results:**

Increased government expenditure on health was significantly and positively correlated with insurance and oral health coverage. It was inversely correlated with out‐of‐pocket expenses (OOPE), private health expenditure, borrowing money to cover health expenses, and ‘All cause’ DALYs (Disability Adjusted Life Years). There was no significant correlation between government health spending and ‘Oral Disorders’ DALYs. Increased private expenditure was inversely correlated with domestic general government health expenditure as a percentage of current health expenditure and oral health coverage and positively correlated with Out‐of‐pocket expenses and borrowing money for covering health expenses. The allocation of government spending did not correspond proportionately to the burden of oral diseases.

**Discussion and Conclusion:**

The lack of correlation between government health funding and the oral disease burden and the disproportionately low government expenditure on oral health relative to the burden of oral diseases when compared to their share of the total disease burden indicates not only a significant deficiency in funding but also misplaced funding priorities. There is a need to focus on the prevention of oral diseases and direct resources towards prevention, regular training, and education of healthcare workers and the public to identify early signs and symptoms of oral disease, rather than solely on treatment.

## Highlights

- Government rather than private spending on health was associated with improved insurance and oral health coverage.
- Increased breadth of oral health coverage was not associated with decreased oral disease burden.
- Inadequate cost sharing and limited spectrum of services offered reduced the effectiveness of oral health coverage.
- Government expenditure on oral health care was not commensurate with the relative burden of oral diseases when compared to the total disease burden.

## 1 Introduction

Oral diseases affect an estimated 3.7 billion people globally, causing pain, discomfort, and disfigurement, and in some cases leading to serious complications or death. They also place a substantial economic burden on individuals and health systems. Despite being largely preventable, these conditions remain widespread. According to the Global Burden of Disease 2021, untreated dental caries is the most common health condition worldwide [1].

The economic impact of dental diseases includes direct costs such as treatment expenses, indirect costs like lost productivity from missing work or school, and intangible costs related to pain and difficulties with eating, speaking, and expressing emotions—factors that affect daily life and social interactions [2]. In 2015, dental diseases were responsible for an estimated $356.8 billion in direct costs and $187.6 billion in indirect costs globally. [3]Dental diseases might also exacerbate the burden of other diseases and thereby contribute to the economic burden of these conditions [3].

Despite all this, oral health remains a low priority in many countries. It is often viewed as an individual responsibility rather than a societal one, leading to its complete or partial exclusion from universal health coverage systems around the world [4]. The global landscape of oral health is shifting with increased recognition of oral diseases and a growing emphasis on policy changes to improve access and quality of care. This includes the World Health Organization's (WHO) Global Action Plan and FDI's Vision 2030, which both aim to improve oral health for all by 2030 and achieve the integration of oral health into Universal Health Coverage (UHC) (WHA74.5) [5]. In fact, achieving UHC for oral health is now the most sought‐after objective under the WHO Strategic Action Plan for Oral Health 2030. It also advocates for timely, comprehensive, and inclusive oral health care within the primary health‐care system [6].

UHC has two main arms that is strengthening primary care and providing financial protection. According to the WHO, health financing is one of the six building blocks essential for a health system to deliver UHC [7]. Sufficient funding enables establishing infrastructure and human resources aiding in providing necessary healthcare, leading to better overall health, while inadequate financing can limit access and contribute to poorer health outcomes. Resource allocation in a healthcare system is influenced by the government priorities and the way funds are allocated within a healthcare system influences which services are prioritized and how resources are distributed.

It has been argued that no country has achieved UHC through voluntary insurance contributions alone. In lower‐middle‐income countries (LMICs), where many people are not formally employed and thus outside income or payroll tax systems, allocating general government revenues is critical. These countries also face major gaps in financial risk protection, making public funding essential to expand coverage [8]. In 2018, out‐of‐pocket expenses on health (OOPE) spending comprised nearly 40% of current health expenditures in LMICs on average [9]. This underscores the critical role of government funding in the successful implementation of UHC.

The Southeast Asian region of the WHO (SEARO) comprising 11 countries that is Bangladesh, Bhutan, Democratic People's Republic of Korea, India, Indonesia, Maldives, Myanmar, Nepal, Sri Lanka, Thailand, and Timor‐Leste is home to a quarter of the world's population where the burden of oral diseases, including dental caries, periodontal diseases, and oral cancers remains high and oral health disparities persist [6]. A recent review on the integration of oral health services into UHC systems in the SEARO region revealed significant challenges. These include limited access to oral health services, uneven distribution of healthcare personnel, poor implementation of oral health policies, poor coverage and inadequate public financing for managing oral diseases [10].

Recognizing the connection between health financing and health outcomes is essential for developing effective policies and interventions that enhance UHC and reduce disease burdens [11]. Correlating government health expenditure in countries with their oral disease burden gives us an idea of the impact (if any) of funding on oral health. Assessing whether government health expenditure allocates funds to oral health in proportion to its share of the overall disease burden can help policymakers and healthcare stakeholders identify potential gaps in oral healthcare financing. The relationship between health expenditure and coverage of dental care in UHC services could give a further idea of where the shortfall lies.

Although there are reports that speak about oral health's neglect and its low priority when it comes to funds allocation and integration into UHC by governments, there are few studies which have tried to study the association between public health financing and oral health outcomes. There is also a scarcity of literature that seeks to quantify the extent of financial/funding shortfall that plagues oral health worldwide.

This study aims to collate the oral health financing landscape, evaluate the relationship between government health expenditure and the burden of oral diseases, assess the proportionality of oral health spending relative to its share of the overall disease burden, and examine the inclusion and funding of dental care within Universal Health Coverage (UHC) benefit packages in the WHO South‐East Asia Region (SEARO).

## 2 Materials and Methods

The reporting was done according to the STROBE guidelines for cross‐sectional studies (Supporting Information S1). This study utilizes publicly available secondary country level data, and no human subjects were involved. As such, ethical approval was not required. However, all data were handled in compliance with ethical research standards, ensuring transparency and accuracy in analysis and reporting.

### 2.1 Sources of Data

Databases comprising health financing and/or health outcomes data of any of the SEARO countries within the past 5 years were reviewed. We sourced data from publicly available databases, including the WHO Regional Health Repository [12], Global Health Expenditure Database (GHED) [13], Global Burden of Disease database (GBD) [14], WHO Oral Health Country/Area Profile Program (CAPP) [15], The Global Findex Database 2021 [16], and OECD (Organization for Economic Co‐operation and Development) (2023) [17].

Additionally, efforts were made to search for papers, documents, books, and relevant national health statistics repositories of SEARO countries to identify any missing data. Indicators or countries were excluded if data were unavailable or older than 5 years. To minimize bias, when more than one dataset was available for a given indicator, we compared them to identify inconsistencies and assess their reliability. We gave preference to sources that use standardized methods across countries, such as those from the World Bank, GBD and WHO. For each variable, we followed a consistent set of criteria to choose among sources, emphasizing those with clear methods and proven accuracy. When national sources offered more recent or detailed data, we cross‐checked them against international figures to maintain consistency. We also standardized the data—aligning units, definitions, and time frames—and made necessary adjustments to ensure the figures were directly comparable.

### 2.2 Health Financing and Disease Burden Indicators

Health finance indicators are statistics that help understand how countries' health systems are funded and how they impact health outcomes [18]. We selected the indicators for our study based on the assumption that these offer a true reflection of health financing information/oral disease burden of the country.

The Global Health Expenditure Database (GHED) [13] offers publicly accessible, comparable data on health expenditures across 194 countries and territories. This database of health finance indicators helps address critical questions, such as the total health spending of countries and territories, the contribution of governments, households, and donors to health funding, and the proportion of spending managed through compulsory or voluntary health financing mechanisms. It is maintained by the WHO and primarily relies on National Health Accounts (NHAs) as the main source for government/private health expenditure data. Additional data is gathered from national sources like ministries of finance, central banks, and statistics offices, as well as international bodies like the World Bank and International Monetary Fund (IMF) [13]. There are 3216 health expenditure indicators in the GHED database. Given the importance of government funding in UHC [8] we (authors) focused on indicators that dealt with government and private share of health expenditure in the SEARO countries.

Domestic general government health expenditure as a percentage of current health expenditure measures the extent to which the government funds healthcare, including transfers, subsidies, and social health insurance contributions [19].Domestic general government health expenditure as a percentage of general government expenditure reflects the proportion of government revenues allocated to health funding [19]. Domestic private health expenditure as a percentage of current health expenditure indicates how much is funded domestically by the private sector [20]. Current health expenditure as a share of GDP (Gross Domestic Product) provides an indication on the level of resources channeled to health relative to other uses [21]. OOPE as a percentage of current health expenditure indicates how much is funded directly by households [22].

Health‐related borrowing underscores the financial strain on individuals and families. ‘Borrowed for health or medical purposes’ is the percentage of respondents who report borrowing any money for health or medical purposes in the past year [23]. Data on the prevalence of borrowing for health or medical purposes was obtained from the World FINDEX database [16].

Health coverage encompasses population coverage (%) eligible for publicly funded health insurance services and ‘oral health coverage’ constitutes the population (%) eligible for publicly funded insurance that covered oral health care [24]. Information on health insurance coverage and oral health coverage among the different countries were obtained from individual studies/published articles specific to each country following a Google search and from the WHO CAPP database [15].

The tax to GDP ratio was obtained from the OECD database [17]. The tax‐to‐GDP ratio measures a country's tax revenue as a percentage of GDP over a specific period. A higher tax to GDP ratio is supposed to illustrate the government's ability to finance its expenditures including health expenditure [25].

A DALY rate is a statistic that measures the overall burden of disease in a population. It is used by the WHO to quantify disease burden. It's a time‐based measure that combines the years of life lost due to premature mortality and years of life lost due to disability. DALY rates are expressed per 100,000 people and are adjusted for age distribution. A higher DALY rate indicates a higher disease burden [26]. The DALY rates for ‘all causes’ and ‘oral disorders’ were obtained from the Global Burden of Disease database (GBD) [14].

### 2.3 Data Extraction and Synthesis

**FIGURE 1:**
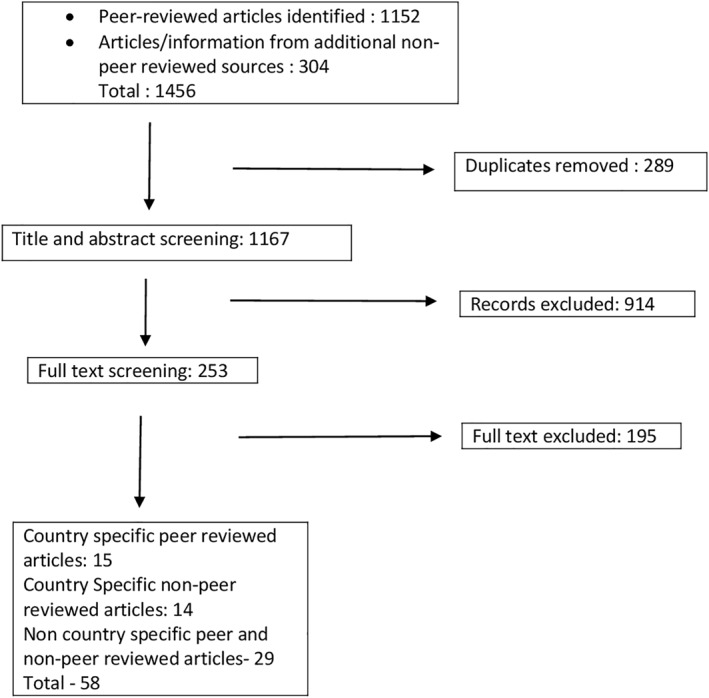
Summary of search, selection and inclusion process (Not including Public Databases).

Data extraction was performed independently by two investigators (SA and MM) using a standardized data extraction form (Supporting Information S2). Search was done with Google using terms like oral health OR dental; Financing OR Funding; AND Health Expenditure AND Universal Health Coverage. Individual SEARO countries were added to these search terms (Supporting Information S3). The strategy was to collect information other than that available in public databases and to check for the latest country data (if any) that was not updated in global databases. Extracted data/information included various health financing and oral health coverage indicators (breadth and depth) for each SEARO country. Findings from included data were summarized to identify common themes, patterns, and discrepancies related to the role of health financing indicators in influencing oral health outcomes across SEARO countries. The percentage of population covered by health insurance/financing schemes as a part of UHC and the extent of dental coverage in these schemes were charted. Information from 10 of the 11 SEARO countries was retrieved from various sources (Figure 1). Democratic People's Republic of Korea was excluded due to dated data (older than 5 years) and missing data that did not facilitate inter‐country comparisons. Descriptive statistics were used to summarize health financing indicators, oral health coverage, and oral disease burden across SEARO countries. Comparative analyses (Pearson correlation) were conducted to examine the relationships between health financing indicators and health outcomes. Statistical analyses were performed using SPSS 29.

## 3 Results

In the WHO South‐East Asia Region (SEARO), oral cancer exhibited the highest DALY rate at 116.04, followed by periodontal diseases (99.5) and edentulism (70.7). Among the countries in the region, Thailand reported the highest overall DALY rate attributable to oral disorders (333.3), followed by Indonesia (282.8) and India (252.8). With regard to oral cancer specifically, India experienced the highest disease burden (138.25), with Sri Lanka ranking second (112.05) [14]. Country‐wise, details of health financing schemes and their coverage of dental care are presented below.

### 3.1 General Health and Oral Health Coverage in SEARO Countries

#### 3.1.1 Bangladesh

The country has no UHC scheme. The Shashtho Shurokkha Karmashuchi (SSK), a healthcare initiative aimed at supporting individuals living below the poverty line (earning < 3 USD/day) covers less than 3% of the population. 15% of the population is covered by Community Based Health Insurance schemes, although dental services remain uncovered by any health financing scheme [27, 28, 29].

#### 3.1.2 Bhutan

The Primary Health Care Scheme (PHC) offers broad access to a range of healthcare services, including essential drugs and treatments, at no cost, and extends coverage to 90% of the population. This includes basic emergency dental care. Within this system, services such as dentures, restorations, crowns and bridges, dental scaling and orthodontic treatment are considered non‐essential and require payment even in government hospitals [30, 31].

#### 3.1.3 India

The Ayushman Bharat—Pradhan Mantri Jan Arogya Yojana (AB‐PMJAY), along with State Government extension schemes, offers comprehensive hospitalization coverage to the bottom uninsured 50% of the population comprising approximately 700 million individuals. Some state schemes cover dental procedures like dentures. Additionally, around 20% of the population, totaling 250 million individuals, benefit from social health insurance and private voluntary health insurance. While some social health insurance schemes, such as the Employee State Insurance scheme for organized employees and the Central Government Health Scheme for government officials, offer limited coverage for dental care, there is no universal dental outpatient coverage for the general population [32, 33].

#### 3.1.4 Indonesia

Jaminan Kesehatan Nasional‐Kartu Indonesia Sehat (JKN‐KIS) is an initiative by the Indonesian government, that aims to provide financial security for every Indonesian citizen in accessing healthcare services [34]. 88% of the population is covered by this scheme. Basic dental health services are included in the insurance coverage, encompassing seven essential services: dental examination, medication, and consultation; premedication; dental emergencies; tooth extraction; post‐extraction medication; dental fillings for disease‐related reasons and not cosmetic; and annual dental scaling [35, 36, 37].

#### 3.1.5 Maldives

Aasandha, the Maldives' national social health insurance initiative, delivers free medical aid to all Maldivian citizens. This scheme operates on full government funding and covers 100% of the population. Within this program, individuals are entitled to receive free basic dental services including routine check‐ups, fillings, extractions, and root canal treatment. However, specialized dental procedures such as orthodontic treatment, crowns and bridges, dentures, or cosmetic enhancements may not fall under the coverage [38, 39, 40].

#### 3.1.6 Myanmar

There are no UHC schemes in Myanmar. Established in 1956, the social security system primarily provides health coverage to employees in Myanmar's formal private sector workforce. However, families of the employees are not covered. This scheme covers less than 2% of Myanmar's population. Furthermore, dental services are not included in this system [41].

#### 3.1.7 Nepal

The National Health Insurance Program (NHIP) is the largest social health insurance scheme and covers 21.4% of the population. Dental services are excluded except primary management of jaw abscess and trauma [42, 43].

#### 3.1.8 Sri Lanka

The government run Agrahara scheme only covers public servants and families. Health services in the public sector are free under the social welfare system and emergency and basic dental care is free for all citizens. School Dental Therapists and Adolescent Dental Clinics deliver free basic oral healthcare and promote oral health among children and adolescents [44, 45, 46].

#### 3.1.9 Thailand

Individuals in Thailand benefit from various insurance schemes, namely the Universal Coverage Scheme (UCS), the Social Security Scheme (SSS), and the Civil Servant Medical Benefits Scheme (CSMBS). These schemes collectively provide comprehensive dental services including oral examinations, scaling, fillings, extractions, and wisdom tooth removal. Under the Universal Coverage Scheme, Thai citizens are entitled to free preventive and curative dental treatments such as fillings, extractions, scaling, plastic prosthesis for deciduous teeth, prosthesis for children with cleft lip and cleft palate, oral health check‐ups, supplemental fluoride for individuals at risk of tooth decay, and dental sealant. More than 98% of the population is covered by these Universal Health Coverage schemes [47, 48].

#### 3.1.10 Timor Leste

Timor‐Leste's health system operates primarily through public financing and provisioning. Medical and dental services are accessible to all without charge at the point of service, resulting in substantial government funding, which constitutes 90% of total healthcare expenses [49, 50].

### 3.2 Descriptive Data on Health Expenditure (Table 1)

Table 1 demonstrates that government health spending as a share of total health expenditure was at its lowest in Bangladesh and Myanmar but reached its peak in Thailand. Conversely, private health expenditure as a proportion of total health spending was highest in Bangladesh and India, while Thailand and Indonesia showed the lowest figures. When it came to domestic general government health spending as a percentage of overall government expenditure, Bangladesh, India, Myanmar, and Nepal ranked the lowest, whereas Thailand ranked the highest, followed by Indonesia and Sri Lanka. OOPE on health was highest in Bangladesh, followed by Myanmar, Nepal, Sri Lanka, and India, but was least in Thailand, with Indonesia coming next among the countries of the SEARO region [13]. India, Sri Lanka, and Bangladesh experienced the highest prevalence of people resorting to borrowing money to cover medical expenses [16]. The tax‐to‐GDP ratio was the highest in Nepal, Maldives and Thailand and lowest in Myanmar and Timor Leste [17].

**TABLE 1:** Health expenditure, coverage and disease burden data from SEARO countries.

	BGD	BTN	IND	IDN	MDV	MMR	NP	LKA	Tha	TLS
Current health expenditure as % gross domestic product (GDP)	2.6	4.4	3.4	3	4.6	5.2	4.1	11.3	4.4	9.9
Domestic general government health expenditure as % gross domestic product (GDP)	0.5	3.4	1.9	1.1	0.7	1.6	1.9	9.1	3.1	5.4
Domestic general government health expenditure as percentage of current health expenditure (%)	16.88	57.43	34.27	59.41	71.6	18.83	33.2	46.45	70.36	63.44
Domestic private health expenditure as percentage of current health expenditure (%)	75.48	20.52	63.52	38.54	15.52	70.31	54.06	49.22	29.5	5.89
Domestic general government health expenditure as percentage of general government expenditure (%)	3.08	5.61	3.69	12.11	18.17	4.39	6.49	9.49	13.4	6.97
Out‐of‐pocket expenditure as percentage of current health expenditure (%)	72.99	18.8	49.82	27.49	14.33	70.25	51.26	43.64	9.04	5.89
Tax/GDP ratio	10.2	10.7	11.7	10.9	17.7	4.4	17.5	10.6	13.4	7.3
Coverage for oral health	0	90	15	88	100	0	0	100	98	100
Health insurance coverage	2.5	90	70	88	100	2	21.3	20	98	1
Borrowed for health or medical purposes	20	2	25	11	10	8	12	24	3	NA
‘Oral disorders’ DALY rates	215.6	227.9	252.8	282.8	239.5	188.5	173.2	173.4	333.3	176.5
‘All cause’ DALY rates	30,475.2	33,001.2	37,843.3	33,996.7	24,579.8	22,219.5	38,863.3	35,952.9	24,227.1	36,370.8
Proportion of oral disorder DALY within all cause DALY rates (%)	0.7	0.69	0.66	0.83	0.97	0.84	0.44	0.48	1.37	0.48
Domestic general government expenditure on dental outpatient curative care, as % of general government expenditure (GGE)	0.00099	0.13292	0.04366	NA	NA	0.00064	NA	NA	0.13894	NA
Dental outpatient curative care, as % of current health expenditure (CHE)	0.20789	0.95513	0.3	NA	NA	0.002795	0.57076	NA	1.52034	NA
Domestic general government expenditure on dental outpatient curative care, as % of current health expenditure (CHE)	0.00596	0.955	0.007	NA	NA	0.002795	NA	NA	0.705	NA
Dental outpatient curative care, in current US$ per capita	0.09901	1.10146	0.048	1.42	1.7	0.00171	0.30155	1.8	4.47677	NA
Domestic general government expenditure on dental outpatient curative care, in current US$ per capita	0.00284	1.10132	0.012	NA	NA	0.00171	NA	NA	2.07598	NA
Domestic private expenditure on dental outpatient curative care, in current US$ per capita	0.09626	NA	0.084775	NA	NA	NA	0.30154	NA	2.4008	NA

*Note:* NA: Data not available.

### 3.3 Health Coverage (Table 1)

Oral health coverage was most comprehensive in Thailand, Sri Lanka, Indonesia and Maldives, with the majority or all the population having access to basic oral health services but was nearly absent in Bangladesh, India, and Nepal. Social health insurance coverage was more widespread in Thailand and Indonesia, followed by India, but lower in Sri Lanka and Nepal, and negligible in Bangladesh and Myanmar [15].

### 3.4 Oral Disease Burden and Oral Health Expenditure (Table 1)

Thailand bore the highest disease burden in terms of DALY rates for both ‘Oral Disorders’ and ‘all causes’ [14]. The proportion of ‘Oral Disorders’ DALY rates within All cause DALY rates (%) ranged from 0.44% in Nepal to 1.37% in Thailand. Some indicator data of oral health expenditure were missing for Indonesia, Maldives, Nepal, Sri Lanka and Timor Leste.

The allocation of government spending did not correspond proportionately to the burden of oral diseases (Oral Disorders DALY rates), especially when compared to its alignment with the overall burden of disease (All cause DALY rates). The proportion of government expenditure on curative dental care was miniscule within the overall dental outpatient curative care as percentage of current health expenditure. The difference in proportion was maximum in Bangladesh and India. Timor Leste, Thailand, and Sri Lanka had the highest per capita expenditure on dental care [13].

### 3.5 Correlates of Health Financing and Coverage Indicators (Table 2)

Table 2 showed that increased government expenditure on health was significantly and positively correlated with insurance and oral health coverage and inversely correlated with OOPE, private health expenditure, prevalence of people borrowing money to cover health expenses and ‘All cause’ DALYs. However, there was no significant correlation between government health spending and Oral DALYs. At the same time, increased private expenditure was negatively correlated with domestic general government health expenditure as percentage of current health expenditure, and oral health coverage and positively correlated with OOPE and iprevalence of borrowing money for covering health expenses. Oral health coverage was positively correlated with ‘All cause’ DALY's but not with ‘Oral Disorders’ DALYs.

**TABLE 2:** Correlation analysis for health expenditure, coverage and disease burden among SEARO countries.

	Domestic general government health expenditure as percentage of current health expenditure (%)	Domestic private health expenditure as percentage of current health expenditure (%)	Domestic general government health expenditure as percentage of general government expenditure (%)	Out‐of‐pocket expenditure as percentage of current health expenditure (%)	UHC coverage for oral health	Health insurance coverage	Borrowed for health or medical purposes	Oral disorders DALY	All cause DALY
Domestic general government health expenditure as percentage of current health expenditure (%)	1	**−0.918** **	**0.791** **	**−0.974** **	**0.811** **	**0.676** *	**−0.701** *	0.380	−0.525
Domestic private health expenditure as percentage of current health expenditure (%)	**−0.918** **	1	−0.594	**0.960** **	**−0.637** *	−0.449	**0.740** *	−0.053	0.328
Domestic general government health expenditure as percentage of general government expenditure (%)	**0.791** **	−0.594	1	**−0.650** *	**0.776** **	0.629	−0.543	0.305	**−0.742** *
Out‐of‐pocket expenditure as percentage of current health expenditure (%)	**−0.974** **	**0.960** **	**−0.650** *	1	**−0.697** *	−0.591	**0.704** *	−0.310	0.384
Coverage for oral health	**0.811** **	**−0.637** *	**0.776** **	**−0.697** *	1	**0.668** *	**−0.833** **	0.508	**−0.744** *
Health insurance coverage	**0.676** *	−0.449	0.629	−0.591	**0.668** *	1	−0.525	0.612	−0.418
Borrowed for health or medical purposes	**−0.701** *	**0.740** *	−0.543	**0.704** *	**−0.833** **	−0.525	1	−0.370	0.542
Oral disorders DALYs	0.380	−0.053	0.305	−0.310	0.508	0.612	−0.370	1	−0.367
All cause DALYs	−0.525	0.328	**−0.742** *	0.384	**−0.744** *	−0.418	0.542	−0.367	1

^*^ *p* ≤ 0.05 Statistically significant.

^**^ *p* ≤ 0.001 Statistically significant.

## 4 Discussion

The aim of our study was to collate the oral health financing landscape, evaluate the relationship between government health expenditure and the burden of oral diseases, assess the proportionality of oral health spending relative to its share of the overall disease burden, and examine the inclusion and funding of dental care within Universal Health Coverage (UHC) benefit packages in the SEARO region. Except for Thailand which is an upper‐middle income country, all the other countries of the SEARO region fall under the lower‐middle income category [13]. We found significant variations in healthcare financing systems and their effectiveness in providing affordable and accessible services to their populations.

### 4.1 Government Funding of Healthcare

Thailand and to some extent, Indonesia had better metrics of government expenditure on health. The low out of pocket expenditure and low private expenditure on health in these countries gives credence to this observation. It was interesting to note that Sri Lanka had a higher proportion of government expenditure on health as compared to India, Myanmar, Nepal and Bangladesh but also had a substantial level of private expenditure. Uneven distribution of government healthcare facilities with inadequate coverage in backward and impoverished areas could be one of the reasons for this [51, 52, 53].

An analysis of health financing indicators showed that higher government spending on health was associated with improved UHC insurance and oral health coverage, lower OOPE, reduced private health spending, lower prevalence of borrowing money to cover healthcare costs, and decreased ‘All cause’ DALYs rates. Earlier studies, employing extensive cross‐national panel datasets, have affirmed the role of increased public funding for healthcare services to enhance health status and achieve UHC. It has been suggested that domestic government health expenditure of at least 5% of GDP is needed to achieve UHC [54, 55, 56]. The average percentage of the SEARO countries for this indicator was 2.8% which is half of the recommended levels.

Reduced government health spending as a percentage of total health expenditure was associated with increased private expenditure on health, higher OOPE, decreased oral health coverage, and increased prevalence of borrowing money for healthcare expenses. Decreased government spending may sideline important social goals such as UHC, allowing the private sector to take the lead, ultimately driving up healthcare costs [57]. A secondary analysis of demographic and health surveys conducted among 70 low‐ and middle‐income countries reported that private services provide about 65% of care for childhood illness [57]. The risks of engaging the private sector need to be strictly managed to prevent market failure in the health sector.^,^ [58, 59] Expanding oral health coverage was associated with a reduction in ‘All cause’ DALYs, but not ‘Oral disorders' DALYs. Oral health coverage might serve as an indicator of overall health coverage. A previous review indicated that enhanced UHC metrics were associated with lower ‘All cause’ DALY rates but not ‘Oral Disorders’ DALY rates [10].

A higher tax to GDP ratio is supposed to illustrate the government's ability to finance its expenditures including health expenditure [25]. While countries with large economy, such as, Thailand can support UHC, countries with smaller GDPs like Nepal demonstrate lower government health expenditure despite having a high tax‐GDP ratio. Nepal also has a higher proportion of private expenditure. A high tax to GDP ratio in a low‐income country can lead to a vicious cycle of lower economic growth and ever higher taxes further slowing economic growth [60].

### 4.2 Oral Health Coverage

Oral health coverage closely followed general health insurance coverage in Thailand, Indonesia, Maldives, Bangladesh, and Myanmar. High oral health coverage was a result of inclusion of dental care in the UHC schemes in Thailand, Indonesia, and Maldives. Low health insurance cover followed low oral health coverage in Bangladesh and Myanmar. The notable exceptions to these were India and Nepal where dental care was not covered comprehensively under the national UHC programs and Sri Lanka where UHC was completely state funded welfare with low penetration of public health insurance coverage. Borrowing money to cover medical expenses was highest in Bangladesh, India and Sri Lanka. Inadequate or no coverage of health insurance, high OOPE in these countries could be the reason. Although the breadth of services (i.e., proportion of population covered) covered by insurance and oral health coverage were the highest in Thailand and Indonesia among the countries of the SEARO, studies have reported a lack of depth in services (variety of services) offered [61, 62, 63]. It is also possible that low private spending in Thailand and Indonesia (i.e., out‐of‐pocket and private insurance) could be the result of barriers to health care [61, 62, 63].

### 4.3 Oral Disease Burden

Thailand and Indonesia bore the highest disease burden in terms of DALY rates. Among the countries in the SEARO region, oral cancer is the leading contributor to DALYs rates for oral diseases, followed by periodontal diseases and edentulism. India has the highest contribution to DALYs from oral cancer, followed by Nepal and Sri Lanka [1, 13]. Although the treatment of oral cancer is included in India's UHC, it is unlikely to significantly reduce DALYs related to oral cancer. This is because most patients seek treatment when the cancer is at an advanced stage with poor prognosis, often after a prolonged period of suffering from the disease.

### 4.4 Challenges

Countries showing optimal oral health coverage and higher government financing may not be providing optimal care. These nations often face significant issues such as limited access to dental services due to uneven distribution and overcrowding of scarce dental facilities (e.g., Sri Lanka) [53], as well as a lack of choice in selecting dental care providers (e.g., Thailand) [62]. Additionally, the funding allocated for dental treatment is often insufficient to cover basic treatments (e.g., Indonesia) [63]. This indicated inadequate government funding for oral health as well as a lack of depth of offered oral care services. This may also explain the high oral disease burden in Thailand [1, 13].

The proportion of “Oral Disorders” DALY rates to ‘All cause’ DALY rates ranged from 0.44% in Nepal to 1.37% in Thailand. However, the government expenditure on oral health care did not follow this proportion. It was lower by a factor of 1000 in Bangladesh and Myanmar, by a factor of 16 in India, by a factor of 10 in Thailand and 5 in Bhutan.

The proportion of government expenditure on dental outpatient curative care, as a percentage of current health expenditure was miniscule. The Domestic Private Expenditure on dental outpatient curative care, in current US$ per capita was almost same as the overall Dental outpatient curative care in current US$ per capita, indicating the large share of private expenditure in dental outpatient care except in Bhutan and Thailand. All health care in Bhutan including oral health care is government sponsored [31], and Thailand has extensive government UCS schemes as well [62]. Consequently, increased government expenditure on health has led to a reduction in All‐cause DALY rates but not Oral‐Disorders DALY rates. This could also be an indicator of oral health not being integrated into general health and being left out.

### 4.5 Opportunities

To effectively reduce DALYs for oral disorders, each country needs to develop strategies tailored to their specific needs and allocate resources to the areas based on their share of contribution to ‘All cause’ DALY rates. All the SEARO countries have a multi‐tiered health care system with primary care facilities offering basic care in rural areas, secondary care facilities in larger towns/cities and tertiary institutions in cities/capitals for advanced care. Most countries have a tax‐based Universal Health Coverage System for dental care, where subsidized or free dental care is offered in public health facilities. Despite the presence of a policy and implementation framework to deliver services, the health care systems, particularly dental care systems, are plagued with manpower, infrastructure issues and extremely shallow depth of services offered [10]. Increased and targeted funding towards mitigating these issues will help in making progress towards achieving the goals of UHC in oral health.

Currently, the limited oral health funding in SEARO countries is predominantly directed towards curative care [10]. However, the focus should shift towards the prevention of oral diseases, especially in countries like India, Sri Lanka, and Bangladesh. Resources should be directed towards prevention, regular training, and education of healthcare workers and the public to identify early signs and symptoms of oral disease, rather than solely on treatment. The United Nations (UN) has prioritized the attainment of UHC as a crucial component of the Sustainable Development Goals (SDG 3.8) within the 2030 Agenda for Sustainable Development in 2015 [64]. Mainstreaming prevention in UHC and fully integrating it with curative care, particularly for oral health, is crucial for controlling costs and effectively addressing the growing burden of oral disease. Prevention is key to oral health [65].

### 4.6 Limitations of the Study

An effort was made to collect data from global databases and not only individual studies to ensure uniformity of criteria and credibility of our findings. This study had several limitations as well. One was the reliance on secondary data that may be subject to reporting biases and inconsistencies across countries. The quality and reliability of data from may also be called into question, as data on disease burden in most SEARO countries comes from anecdotal studies with smaller sample sizes. It is also possible that different agencies within a single country can report varying datasets due to differences in methodology, definitions, reporting periods, or political influences.

Considering the possible lack of harmony between different data sources We can say that the associations observed in our study need to be further studied, possibly with a larger dataset comprising more countries. We excluded the Democratic Republic of Korea from our analysis due to dated and incomplete data which did not facilitate comparison. Specific oral health expenditure related data was not available for some of the SEARO countries which precluded their correlation with other indicators. The use of various data sources can be a weakness but is also a strength of the paper as this is the first attempt to collate the oral health financing landscape in SEARO countries. The intention of this paper was to point attention to the possible role of health financing in influencing oral health coverage based on currently available data and to stimulate further research in this area.

## 5 Conclusion

We found that financing oral health services through integration into general health systems remains a work in progress in SEARO countries. Current financing models of UHC have largely neglected oral health. The lack of correlation between government health funding and the oral disease burden could indicate either a significant deficiency in funding or misplaced funding priorities or a problem with the quality of data. There is an overemphasis on curative care at the expense of preventive and promotive efforts, leading to the isolation of oral health in the general scheme of UHC [10]. Increased government spending will be effective only if oral health is integrated into general health systems, or if oral health systems are aligned with general health systems. Therefore, instead of considering oral health services in isolation, efforts should be made to include oral health as part of the essential health care package. We do not need a separate essential package for oral health but an integrated essential health care package that includes oral health as a component. These findings indicate the multitude of interactions between economic, political, and social factors shaping healthcare systems and outcomes in the SEARO region. Addressing disparities in health spending, coverage, and outcomes requires a multifaceted approach involving policy reforms, resource mobilization, and strategic investments in healthcare infrastructure and services.

## Ethics Statement

The authors have nothing to report.

## Conflicts of Interest

The authors declare no conflicts of interest.

## Supporting information


Supporting Information S1

Supporting Information S2

Supporting Information S3

## Data Availability

The data that support the findings of this study are available in Global Health Expenditure Database at https://apps.who.int/nha/database. These data were derived from the following resources available in the public domain: ‐ World Health Organization, https://apps.who.int/nha/database ‐ Institute of Health Metrics and Evaluation, https://www.healthdata.org/.

## References

[1] M. A. Peres , L. M. D. Macpherson , R. J. Weyant , et al., “Oral Diseases: A Global Public Health Challenge,” Lancet 394, no. 10194 (2019): 249–260, 10.1016/S0140-6736(19)31146-8. 31327369

[2] S. Listl , J. Galloway , P. A. Mossey , and W. Marcenes , “Global Economic Impact of Dental Diseases,” Journal of Dental Research 94, no. 10 (2015): 1355–1361, 10.1177/0022034515602879. 26318590

[3] A. J. Righolt , M. Jevdjevic , W. Marcenes , and S. Listl , “Global‐regional‐and Country‐Level Economic Impacts of Dental Diseases in 2015,” Journal of Dental Research 97, no. 5 (2018): 501–507, 10.1177/0022034517750572. 29342371

[4] T. T. Wang , M. R. Mathur , and H. Schmidt , “Universal Health Coverage, Oral Health, Equity and Personal Responsibility,” Bulletin of the World Health Organization 98, no. 10 (October 2020): 719–721, 10.2471/BLT.19.247288. 33177761 PMC7652557

[5] World Health Organization . “Seventy‐Fourth World Health Assembly,” Oral Health (May 2021): Agenda item 13.2, https://apps.who.int/gb/ebwha/pdf_files/wha74/a74_r5‐en.pdf.

[6] Action Plan for Oral Health in South‐East Asia 2022–2030: Towards Universal Health Coverage for Oral Health. (World Health Organization, Regional Office for South‐East Asia; 2022). Accessed April.2023, https://www.who.int/publications‐detail‐redirect/9789290210061.

[7] World Health Organization . Monitoring the Building Blocks of Health Systems: A Handbook of Indicators and Their Measurement Strategies (World Health Organization, 2010), https://iris.who.int/handle/10665/258734.

[8] J. Kutzin and World Health Organisation . “Anything Goes on the Path to Universal Health Coverage?,” (2012), Accessed 16 March 2024, http://www.who.int/bulletin/volumes/90/11/12‐113654/en/. 10.2471/BLT.12.113654 PMC3506412 23226900

[9] World Bank . World Development Indicators, Accessed 5 Nov 2021, https://databank.worldbank.org/source/world‐development‐indicators.

[10] S. Acharya , M. R. Mathur , S. K. Tadakamadla , and A. Brand , “Assessing the Status of Oral Health Integration in South East Asian Regional Office Countries' Universal Health Coverage‐A Scoping Review,” International Journal of Health Planning and Management 39, no. 2 (March 2024): 262–277, 10.1002/hpm.3751. 38169038

[11] World Health Organization , “Health Topics,” Health Financing, https://www.who.int/health‐topics/health‐financing#tab=tab_1.

[12] World Health Organization , Regional Health Repository, accessed February 2024, https://www.who.int/data/gho.

[13] Global Health Expenditure Database. World Health Organization. Accessed February 2024, https://apps.who.int/nha/database/Select/Indicators/en.

[14] Global Burden of Disease Collaborative Network , Global Burden of Disease Study 2019 (Institute for Health Metrics and Evaluation (IHME), 2020), Accessed September.2023, https://www.healthdata.org/research‐analysis/gbd.

[15] “WHO Oral Health Country/Area Profile Program,” accessed January 2024, https://capp.mau.se/about‐capp/.

[16] “The Global Findex Database,” (2021). accessed February 2024, https://www.worldbank.org/en/publication/globalfindex/Data.

[17] Revenue Statistics in Asia and the Pacific 2023: Strengthening Property Taxation in Asia, OECD Publishing, accessed February 2024, 10.1787/e7ea496f-en.

[18] National Health Profile , An Overview of Public Health Expenditure Including Pattern of Allocations Made to Central/State Governments Etc (Central Bureau of Health Intelligence. Ministry of Health and Family Welfare, Government of India, 2018), https://cbhidghs.mohfw.gov.in/WriteReadData/l892s/Chapter%204.pdf.

[19] WHO. Global Health Observatory , Domestic General Government Health Expenditure (GGHE‐D) as Percentage of Current Health Expenditure (CHE) (%), https://www.who.int/data/gho/indicator‐metadata‐registry/imr‐details/4953.

[20] WHO. Global Health Observatory , Domestic Private Health Expenditure (PVT‐D) as Percentage of Current Health Expenditure (CHE) (%), https://www.who.int/data/gho/indicator‐metadata‐registry/imr‐details/4954.

[21] WHO. Global Health Observatory , Current Health Expenditure (CHE) as Percentage of Gross Domestic Product (GDP) (%), https://www.who.int/data/gho/indicator‐metadata‐registry/imr‐details/4950.

[22] WHO. Global Health Observatory , Out‐of‐pocket Expenditure as Percentage of Current Health Expenditure (CHE) (%), https://www.who.int/data/gho/indicator‐metadata‐registry/imr‐details/4965.

[23] World Bank , Global Findex Glossary, https://www.worldbank.org/content/dam/Worldbank/Research/GlobalFindex/PDF/Glossary.pdf.

[24] International Standard Methodology (A System of Health Accounts SHA 2011), https://www.who.int/publications/i/item/9789240042551, https://www.who.int/health‐topics/health‐accounts.

[25] C. Pessino and R. Fenochietto , “Determining Countries' Tax Effort,” Hacienda Publica Espanola 195 (2010): 65–87, https://www.researchgate.net/publication/227439705_Determining_Countries'_Tax_Effort.

[26] WHO , Global Health Estimates: Leading Causes of DALYs, https://www.who.int/data/gho/data/themes/mortality‐and‐global‐health‐estimates/global‐health‐estimates‐leading‐causes‐of‐dalys.

[27] Md Zahid Hasan , S. Ahmed , G. M. Gazi , M. W. Ahmed , S. El Arifeen , and M. E. Chowdhury , “The Effectiveness of a Government‐Sponsored Health Protection Scheme in Reducing Financial Risks for the Below‐Poverty‐Line Population in Bangladesh,” Health Policy and Planning (2023): czad115, 10.1093/heapol/czad115. PMC11423846 38164712

[28] “Can Bangladesh Achieve Universal Health Coverage? Writer : Shabnam Mostari and Nusrat Parvin Mohona Source : Dhaka Tribune,” (September 2023). accessed February 2024, https://a2i.gov.bd/can‐bangladesh‐achieve‐universal‐health‐coverage/#:~:text=With%20only%202.5%25%20of%20the,coverage%20(UHC)%20by%202032.

[29] Ministry of Health and Family Welfare , “Government of the People's Republic of Bangladesh. accessed February 2024, http://www.mohfw.gov.bd/index.php?option=com_content&view=article&id=166&Itemid=150&lang=bn.

[30] Government of Bhutan , National Health Policy, (2009). accessed January 2024, http://www.health.gov.bt/wp‐content/uploads/moh‐files/2015/11/National‐Health‐Policy.pdf.

[31] K. Tenzin , T. Dorji , G. Dorji , and D. E. Lucero‐Prisno III , “Lucero‐Prisno III DE. Health Inequities in Bhutan's Free Healthcare System: A Health Policy Dialogue Summary,” Public Health Chall 1, no. 4 (2022): e34, 10.1002/puh2.34. 40496697 PMC12039577

[32] Kumar A. , and S. Rakesh , “Health Insurance for India’s Missing Middle Publishing Agency: NITI Aayog Year of Publication,” (2021) Book, English, 10.31219/osf.io/s2x8r

[33] Priyanka Sharma , Premium Rates for PM‐JAY to Rise for the First Time. accessed February 2024, https://www.livemint.com/news/india/nha‐plans‐to‐revise‐premium‐for‐world‐s‐largest‐health‐assurance‐scheme‐pradhan‐mantri‐jan‐arogya‐yojana‐ab‐pm‐jay‐in‐new‐delhi‐11685553739511.html.

[34] R. A. Fattah , Q. Cheng , H. Thabrany , et al., “Incidence of Catastrophic Health Spending in Indonesia: Insights From a Household Panel Study 2018‐2019,” International Journal for Equity in Health 22, no. 1 (2023): 185, 10.1186/s12939-023-01980-w. 37674199 PMC10483778

[35] “Number of Beneficiaries of the National Health Insurance (JKN‐KIS) in Indonesia From 2014 to 2022,” accessed February 2024, https://www.statista.com/statistics/1154704/indonesia‐number‐of‐jkn‐kis‐beneficiaries/.

[36] JKN Policyholders Hope for Fair, Affordable Premiums Under Single Class Care,” Jakarta Post 18. no. 5.(2024), https://www.thejakartapost.com/indonesia/2024/05/18/jkn‐policyholders‐hope‐for‐fair‐affordable‐premiums‐under‐single‐class‐care.html#:~:text=First%2D%20and%20s%2Dclass%20policyholders,7%2C000%20subsidy%20from%20the%20government.

[37] N. Hariyani , D. Setyowati , M. R. Sari , D. A. Maharani , R. Nair , and K. Sengupta , “Factors Influencing the Utilization of Dental Services in East Java, Indonesia,” F1000Res 9 (July 2020): 673, 10.12688/f1000research.23698.1. 33968372 PMC8082568

[38] National Social Protection Agency , Male. Social Health Insurance Scheme (Aasandha). accessed February 2024, https://www.nspa.gov.mv/v2/index.php/aasandha‐2/.

[39] H. Faiz , “Universal Health Insurance Scheme in Maldives,”(August 2023), https://www.linkedin.com/pulse/universal‐health‐insurance‐scheme‐maldives‐hamdhoon‐faiz/.

[40] “Healthcare and Insurance in Maldives,” accessed January 2024, https://www.uscisguide.com/international/healthcare‐and‐insurance‐in‐maldives/.

[41] Universal Health Coverage Moving Toward , Myanmar. National Initiatives, Key Challenges, and the Role of Collaborative Activities, accessed February 2024, https://documents.worldbank.org/curated/en/991991513148339321/pdf/122045‐BRI‐Moving‐Toward‐UHC‐series‐PUBLIC‐WorldBank‐UHC‐Myanmar‐FINAL‐Nov30.pdf.

[42] G. N. Khanal , B. Bharadwaj , N. Upadhyay , T. Bhattarai , M. Dahal , and R. B. Khatri , “Evaluation of the National Health Insurance Program of Nepal: Are Political Promises Translated into Actions?,” Health Research Policy and Systems 21, no. 1 (January 2023): 7, 10.1186/s12961-022-00952-w. 36670433 PMC9862822

[43] S. Deo , S. Shrestha , S. Gupta , et al., “Health Insurance for Equitable Healthcare Services in Nepal: A Situation Analysis,” JNDA 18: (2018)No. 1 (January ‐ June 2018): 42‐4National Insurance Trust Fund. accessed January 2024, https://www.nitf.lk/en/insurance.html.

[44] Budget Brief: Health Sector Sri Lanka 2021. accessed February 2024, https://www.unicef.org/srilanka/media/2716/file/BUDGET%20BRIEF:%20HEALTH%20SECTOR%202021.pdf.

[45] M. N. Karunatilaka and S. M. Samarage , “Knowledge of “Agrahara” Medical Insurance Scheme Among Employees of a Selected Public Sector Institution in Sri Lanka,” International Journal of Scientific and Research Publications 10, no. 9 (September 2020): 912–916, 10.29322/ijsrp.10.09.2020.p105111.

[46] World Health Organization , Oral Health Sri Lanka 2022 Country Profile, accessed January 2024, https://www.who.int/publications/m/item/oral‐health‐lka‐2022‐country‐profile.

[47] K. Srimuang and P. Pholphirul , “Moral Hazard and the Demand for Dental Treatment: Evidence From a Nationally Representative Survey in Thailand,” Int J Dent 2022, no. 1 (August 2022): 2259038, 10.1155/2022/2259038. 36034478 PMC9402366

[48] World Bank Group , “Thailand Public Revenue and Spending Assessment. Promoting an Inclusive and Sustainable Future,” accessed February 2024, https://documents1.worldbank.org/curated/en/099052523201027923/pdf/P1771570af8b360b40aab305f0be65a2678.pdf.

[49] L. Guinness , R. C. Paul , J. S. Martins , et al., “Determinants of Health Care Utilisation: The Case of Timor‐Leste,” Int Health 10, no. 6 (November 2018): 412–420, 10.1093/inthealth/ihy044. 30007293 PMC6204763

[50] International Labour Organization , “Timor‐Leste to Extend its Social Security Coverage to Informal Workers,” (August 2023), accessed February, 2024, https://www.ilo.org/jakarta/info/public/pr/WCMS_890730/lang‐‐en/index.htm#:~:text=To%20date%2C%20in%20Timor%2DLeste,the%20Pacific%20countries%20(44.1%25.

[51] O. Basnayake and I. Perera , “Oral Health: A Review through Eyes of Sri Lanka,” International Journal of Scientific and Research Publications (IJSRP) 11, no. 4 (2021): 508–511, 10.29322/IJSRP.11.04.2021.p11268.

[52] Y. A. Jayasinghe , S. M. Jayawickrama , S. Ratnapreya , R. M. Jayasinghe , D. De Silva , and R. D. Jayasinghe , “Challenges Faced in Dental Care Delivery amid Financial Crisis in Sri Lanka: An Evidence‐Based Analysis From the Perspective of Health Professionals,” Inside Business 3, no. 4 (2023): 524–533, 10.3390/businesses3040032.

[53] I. Perera , E. Kruger , and M. Tennant , “GIS as a Decision Support Tool in Health Informatics: Spatial Analysis of Public Dental Care Services in Sri Lanka,” Journal of Health Informatics in Developing Countries 6 (2012): 422–433, https://www.jhidc.org/index.php/jhidc/article/view/82.

[54] D. Mcintyre , F. Meheus , and J. A. Røttingen , “What Level of Domestic Government Health Expenditure Should We Aspire to for Universal Health Coverage?,” Health Economics, Policy and Law 12, no. 2 (April 2017): 125–137: PMID: 28332456, 10.1017/S1744133116000414. 28332456

[55] R. Moreno‐Serra and P. Smith , “Broader Health Coverage Is Good for the Nation’s Health: Evidence From Country Level Panel Data,” Journal of the Royal Statistical Society 178, no. 1 (2015): 101–124, 10.1111/rssa.12048. PMC4280714 25598588

[56] R. Reeves , Y. Gourtsoyannis , S. Basu , D. McCoy , M. McKee , and D. Stuckler , “Financing Universal Health Coverage – Effects of Alternative Tax Structures on Public Health Systems: Cross‐National Modelling in 89 Low‐Income and Middle‐Income Countries,” Lancet 386, no. 9990 (2015): 274–280, 10.1016/s0140-6736(15)60574-8. 25982041 PMC4513966

[57] K. A. Grepin , “Private Sector an Important but Not Dominant Provider of Key Health Services in Low‐ and Middle‐Income Countries,” Health Affairs 35, no. 7 (2016): 1214–1221, 10.1377/hlthaff.2015.0862. 27385236

[58] WHO. Technical Series on Primary Health Care , The Private Sector, Universal Health Coverage and Primary Health Care (WHO/HIS/SDS/2018.53 © World Health Organization, 2018), Accessed February 2024, https://www.who.int/publications/i/item/WHO‐HIS‐SDS‐2018.53.

[59] T. E. Collins , S. Akselrod , L. Mahy , et al., “Engaging With the Private Sector for Noncommunicable Disease Prevention and Control: Is it Possible to Create ‘Shared Value’?,” Annals of Global Health 89, no. 1 (July 2023): 46, 10.5334/aogh.4136. 37425141 PMC10327866

[60] A. Dahal , “Tax‐to‐GDP Ratio and the Relation of Tax Revenue With GDP: Nepalese Perspective. Researcher,” A Research Journal of Culture and Society 4, no. 1 (2020): 80–96, 10.3126/researcher.v4i1.33813.

[61] R. Namwichaisirikul , N. Pudpong , and W. Panichkriangkrai , “Analysis of Dental Service Utilization and Dental Public Health Policy Among Thai Population in A Past Decade,” Khon Kaen Dent J [Internet] 21, no. 2 (December 2018): 178: [cited 2023 Aug. 12]. Accessed on 12.05.23, https://he01.tci‐thaijo.org/index.php/KDJ/article/view/163709.

[62] N. Chaianant , T. Tussanapirom , M. Kettratad , et al., “Inequalities in Dental Service Utilization Among Thai Adults From 2000 to 2017,” Community Dentistry and Oral Epidemiology 51, no. 4 (August 2023): 660–670, 10.1111/cdoe.12884. 37350457

[63] I. Dewanto , S. Koontongkaew , and N. Widyanti , “Adequacy of Dental Capitation Payment at Community Health Centers in the Implementation of Indonesian National Health Insurance,” Journal of International Oral Health 13, no. 3 (2021): 274–280, 10.4103/jioh.jioh_311_20.

[64] M. Mackintosh and M. Koivusalo , “Health Systems and Commercialization: In Search of Good Sense,” in Commercialization of Health Care, editer by M. Mackintosh and M. Koivusalo . (Palgrave Macmillan, 2005).

[65] Editorial: Oral Health: Prevention Is Key”. Lancet. (2009);373, no. 9657: 1, 10.1016/S0140-6736(08)61933-9. 19121705

