# Supplementary material for: Linking Health Financing to Oral Health Coverage and Disease Burden in SEARO Countries: A Cross‐Sectional Analysis of Country Level Data
Source: Int J Health Plann Manage. 2025 Sep 30;41(1):47–58. doi: 10.1002/hpm.70027 (PMC12794124; doi:10.1002/hpm.70027)
Supplement: Supplementary file 2 — Supporting Information S2 [file HPM-41-47-s003.docx]

 Supplement 2 - Data extraction template for Health expenditure, disease burden and coverage data among the SEARO countries.

| **Country** | BGD | BTN | IND | IDN | MDV | MMR | NP | LKA | THA | TLS |
| --- | --- | --- | --- | --- | --- | --- | --- | --- | --- | --- |
| Current Health Expenditure as % Gross Domestic Product (GDP) |  |  |  |  |  |  |  |  |  |  |
| Domestic General Government Health Expenditure as % Gross Domestic Product (GDP) |  |  |  |  |  |  |  |  |  |  |
| Domestic general government health expenditure as percentage of current health expenditure (%) |  |  |  |  |  |  |  |  |  |  |
| Domestic private health expenditure as percentage of current health expenditure (%) |  |  |  |  |  |  |  |  |  |  |
| Domestic general government health expenditure as percentage of general government expenditure (%) |  |  |  |  |  |  |  |  |  |  |
| Out-of-pocket expenditure as percentage of current health expenditure (%) |  |  |  |  |  |  |  |  |  |  |
| tax/GDP ratio |  |  |  |  |  |  |  |  |  |  |
| Coverage for oral health |  |  |  |  |  |  |  |  |  |  |
| Health Insurance coverage |  |  |  |  |  |  |  |  |  |  |
| Borrowed for health or medical purposes |  |  |  |  |  |  |  |  |  |  |
| ‘Oral disorders’ DALY rates |  |  |  |  |  |  |  |  |  |  |
| ‘All cause’ DALY rates |  |  |  |  |  |  |  |  |  |  |
| Dominant UHC Scheme |  |  |  |  |  |  |  |  |  |  |
| Domestic General Government Expenditure on Dental outpatient curative care, as % of general government expenditure (GGE) |  |  |  |  |  |  |  |  |  |  |
| Dental outpatient curative care, as % of current health expenditure (CHE) |  |  |  |  |  |  |  |  |  |  |
| Domestic General Government Expenditure on Dental outpatient curative care, as % of current health expenditure (CHE) |  |  |  |  |  |  |  |  |  |  |
| Dental outpatient curative care, in current US$ per capita |  |  |  |  |  |  |  |  |  |  |
| Domestic General Government Expenditure on Dental outpatient curative care, in current US$ per capita |  |  |  |  |  |  |  |  |  |  |
| Domestic Private Expenditure on Dental outpatient curative care, in current US$ per capita |  |  |  |  |  |  |  |  |  |  |

BGD: Bangladesh, BTN: Bhutan, IND: India, IDN: Indonesia, MDV: Maldives, MMR: Myanmar, NP: Nepal, LKA : Sri Lanka, THA: Thailand, TLS: Timor Leste

Source (Options)

-Systematic review

-Scoping review

-Narrative review

- Intervention study

-Observational study

- Non peer reviewed articles

-Grey literature

-Website

-Government/Policy/WHO and other multilateral organizations’ documents
