# Supplementary material for: Linking Health Financing to Oral Health Coverage and Disease Burden in SEARO Countries: A Cross‐Sectional Analysis of Country Level Data
Source: Int J Health Plann Manage. 2025 Sep 30;41(1):47–58. doi: 10.1002/hpm.70027 (PMC12794124; doi:10.1002/hpm.70027)
Supplement: Supplementary file 3 — Supporting Information S3 [file HPM-41-47-s001.docx]

**Search Strategy :**

Search:

(((((oral health) OR (dental)) AND (financing)) OR (funding)) AND (health expenditure)) AND (universal health coverage)

((("oral health"[MeSH Terms] OR ("oral"[All Fields] AND "health"[All Fields]) OR "oral health"[All Fields] OR ("dental health services"[MeSH Terms] OR ("dental"[All Fields] AND "health"[All Fields] AND "services"[All Fields]) OR "dental health services"[All Fields] OR "dental"[All Fields] OR "dentally"[All Fields] OR "dentals"[All Fields])) AND ("economics"[MeSH Subheading] OR "economics"[All Fields] OR "finances"[All Fields] OR "economics"[MeSH Terms] OR "financing"[All Fields] OR "finance"[All Fields] OR "financed"[All Fields] OR "financer"[All Fields] OR "financers"[All Fields] OR "financings"[All Fields])) OR ("economics"[MeSH Subheading] OR "economics"[All Fields] OR "funding"[All Fields] OR "economics"[MeSH Terms] OR "financial management"[MeSH Terms] OR ("financial"[All Fields] AND "management"[All Fields]) OR "financial management"[All Fields] OR "funded"[All Fields] OR "funds"[All Fields] OR "fund s"[All Fields] OR "fundings"[All Fields])) AND ("health expenditures"[MeSH Terms] OR ("health"[All Fields] AND "expenditures"[All Fields]) OR "health expenditures"[All Fields] OR ("health"[All Fields] AND "expenditure"[All Fields]) OR "health expenditure"[All Fields]) AND (("universal"[All Fields] OR "universalism"[All Fields] OR "universalities"[All Fields] OR "universality"[All Fields] OR "universalization"[All Fields] OR "universalize"[All Fields] OR "universalized"[All Fields] OR "universalizing"[All Fields] OR "universally"[All Fields] OR "universals"[All Fields] OR "universe"[All Fields] OR "universes"[All Fields]) AND ("health"[MeSH Terms] OR "health"[All Fields] OR "health s"[All Fields] OR "healthful"[All Fields] OR "healthfulness"[All Fields] OR "healths"[All Fields]) AND ("coverage"[All Fields] OR "coverages"[All Fields]))

**((((((((((((((((oral health) OR (dental)) AND (financing)) OR (funding)) AND (health expenditure)) AND (universal health coverage)) OR (India)) OR (Indonesia)) OR (Maldives)) OR (Nepal)) OR (Thailand)) OR (Myanmar)) OR (Timor leste)) OR (Sri lanka)) OR (Bangladesh)) OR (Bhutan)) OR (DPRK)**

((("oral health"[MeSH Terms] OR ("oral"[All Fields] AND "health"[All Fields]) OR "oral health"[All Fields] OR ("dental health services"[MeSH Terms] OR ("dental"[All Fields] AND "health"[All Fields] AND "services"[All Fields]) OR "dental health services"[All Fields] OR "dental"[All Fields] OR "dentally"[All Fields] OR "dentals"[All Fields])) AND ("economics"[MeSH Subheading] OR "economics"[All Fields] OR "finances"[All Fields] OR "economics"[MeSH Terms] OR "financing"[All Fields] OR "finance"[All Fields] OR "financed"[All Fields] OR "financer"[All Fields] OR "financers"[All Fields] OR "financings"[All Fields])) OR ("economics"[MeSH Subheading] OR "economics"[All Fields] OR "funding"[All Fields] OR "economics"[MeSH Terms] OR "financial management"[MeSH Terms] OR ("financial"[All Fields] AND "management"[All Fields]) OR "financial management"[All Fields] OR "funded"[All Fields] OR "funds"[All Fields] OR "fund s"[All Fields] OR "fundings"[All Fields])) AND ("health expenditures"[MeSH Terms] OR ("health"[All Fields] AND "expenditures"[All Fields]) OR "health expenditures"[All Fields] OR ("health"[All Fields] AND "expenditure"[All Fields]) OR "health expenditure"[All Fields]) AND (("universal"[All Fields] OR "universalism"[All Fields] OR "universalities"[All Fields] OR "universality"[All Fields] OR "universalization"[All Fields] OR "universalize"[All Fields] OR "universalized"[All Fields] OR "universalizing"[All Fields] OR "universally"[All Fields] OR "universals"[All Fields] OR "universe"[All Fields] OR "universes"[All Fields]) AND ("health"[MeSH Terms] OR "health"[All Fields] OR "health s"[All Fields] OR "healthful"[All Fields] OR "healthfulness"[All Fields] OR "healths"[All Fields]) AND ("coverage"[All Fields] OR "coverages"[All Fields]))) OR ("india"[MeSH Terms] OR "india"[All Fields] OR "india s"[All Fields] OR "indias"[All Fields]) OR ("indonesia"[MeSH Terms] OR "indonesia"[All Fields] OR "indonesia s"[All Fields] OR "indonesias"[All Fields]) OR ("maldive"[All Fields] OR "maldives"[MeSH Terms] OR "maldives"[All Fields]) OR ("nepal"[MeSH Terms] OR "nepal"[All Fields] OR "nepal s"[All Fields]) OR ("thailand"[MeSH Terms] OR "thailand"[All Fields] OR "thailand s"[All Fields]) OR ("myanmar"[MeSH Terms] OR "myanmar"[All Fields] OR "myanmar s"[All Fields] OR "myanmars"[All Fields]) OR ("timor leste"[MeSH Terms] OR "timor leste"[All Fields] OR ("timor"[All Fields] AND "leste"[All Fields]) OR "timor leste"[All Fields]) OR ("sri lanka"[MeSH Terms] OR ("sri"[All Fields] AND "lanka"[All Fields]) OR "sri lanka"[All Fields]) OR ("bangladesh"[MeSH Terms] OR "bangladesh"[All Fields] OR "bangladesh s"[All Fields]) OR ("bhutan"[MeSH Terms] OR "bhutan"[All Fields] OR "bhutan s"[All Fields]) OR "DPRK"[All Fields]
